# Efficient enrichment of plasma-derived extracellular vesicles from small volumes of bovine blood

**DOI:** 10.1093/jas/skaf354

**Published:** 2025-10-15

**Authors:** Vincent Prieur, Isabelle Cassar-Malek, Arnaud Delavaud, Liam Barry-Carroll, Jean-Christophe Delpech, Isabelle Morel, Sylvain Lerch, Didier Viala, Philip Chennell, Céline Boby, Muriel Bonnet

**Affiliations:** INRAE, Université Clermont Auvergne, VetAgro Sup, UMR Herbivores, Saint-Genès-Champanelle, F-63122, France; INRAE, Université Clermont Auvergne, VetAgro Sup, UMR Herbivores, Saint-Genès-Champanelle, F-63122, France; INRAE, Université Clermont Auvergne, VetAgro Sup, UMR Herbivores, Saint-Genès-Champanelle, F-63122, France; University of Bordeaux, INRAE, Bordeaux INP, NutriNeuro, UMR 1286, Bordeaux, F-33000, France; University of Bordeaux, INRAE, Bordeaux INP, NutriNeuro, UMR 1286, Bordeaux, F-33000, France; Ruminant Nutrition and Emissions, Agroscope, 1725 Posieux, Switzerland; Ruminant Nutrition and Emissions, Agroscope, 1725 Posieux, Switzerland; INRAE, Université Clermont Auvergne, VetAgro Sup, UMR Herbivores, Saint-Genès-Champanelle, F-63122, France; PlateForme d‘Exploration du Métabolisme, Composante Protéomique (PFEMcp), INRAE, Centre Clermont-Auvergne-Rhône-Alpes, Saint-Genès-Champanelle, 63122, France; Université Clermont Auvergne, CHU Clermont Ferrand, Clermont Auvergne INP, CNRS, ICCF, Clermont-Ferrand, F-63000, France; INRAE, Université Clermont Auvergne, VetAgro Sup, UMR Herbivores, Saint-Genès-Champanelle, F-63122, France; INRAE, Université Clermont Auvergne, VetAgro Sup, UMR Herbivores, Saint-Genès-Champanelle, F-63122, France

**Keywords:** Biomarker discovery, lipoprotein removal, methodology optimization, phenotypic expressions, proteomics, vesicle-associated proteins

## Abstract

There is a growing interest in small extracellular vesicles (sEVs). These nanoparticles, which range in diameter from 30 to 150 nm, are secreted by cells into their surrounding environment and transfer biological content to distant cells. However, the lack of consensus on sEV isolation, from bovine plasma limits their study. This work aimed to develop an optimized method to enrich sEVs from 4 mL of bovine blood plasma. To increase the yield of sEVs while reducing contamination from other particles and free proteins, sEVs were isolated from 38 bovine plasma samples of crossbred heifers using sequential centrifugation and filtration with size-exclusion chromatography. In accordance with the Minimal Information for Studies of Extracellular Vesicles (MISEV) guidelines, the sEV preparations were characterized in terms of size, particles concentration, morphology, and sEV markers. To accurately estimate particle size and distribution, we used a combination of three methods. This approach confirmed that 76% of the particles fell within the expected range of 30-150 nm for sEVs. The preparations were pure, with an average particle-to-protein ratio of 2.4 × 10^8^ particles/µg of protein. This is comparable to or exceeds recent observations in bovine and other mammalian species when blood plasma and serum are used. Moreover, albumin, accounted for only 1.8–6.5% of the final protein abundance, indicating a 90–98% depletion relatively to raw plasma. Microscopy confirmed the presence of cup-shaped particles characteristic of sEVs. Proteomic characterization identified 417 proteins (FDR 1%, ≥ 2 peptides), corresponding to 372 unique homologous human gene names, including the cytosolic (HSPA8, SDCBP, ACT, TUB, GAPDH) and membrane (CD9, CD81) markers of sEVs. Of these proteins, 347 (93%) are referenced in  Vesiclepedia, an international database of sEV proteome, suggesting a strong enrichment of sEVs during the purification process. This finding is supported by the identification of 172 significantly enriched Gene Ontology terms related to sEV annotation (*P *< 0.01, Fisher’s one-tailed test with Benjamin–Hochberg correction) such as GO:0005615 (extracellular space) and GO:1903561 (extracellular vesicle). According to the MISEV guidelines and proteomic requirements, the proposed optimized sEV enrichment protocol is suitable for 4 mL of plasma. These results pave the way for future research into the role of sEVs in relation to animal health and performance.

## Teaser

We have optimized a protocol for isolating small extracellular vesicles (sEVs) from 4 mL of bovine plasma. We achieved an average particle-to-protein ratio of 2.4 × 10^8^ particles/µg of protein, with the presence of sEVs confirmed by morphological and molecular features, while reducing the required plasma volume by 70%–90% from original protocol.

## Introduction

Extracellular vesicles (EVs) are nanovesicles (30–150 nm) ubiquitous in mammalian biofluids composed of 2 main subgroups: exosomes (30–150 nm), which are secreted through the endosomal pathway (Blandin and Le Lay, 2020; Gurung et al., 2021), and small microvesicles (also named ectosomes, 100–1000 nm), which bud directly from the plasma membrane. Small EVs (sEVs), whom the size in under 150 nm, are essential for inter-organ communication. They have the ability to carry between tissues and organs biological material, including nucleic acids, proteins, and lipids. Consequently, they can reflect the metabolic status of organisms (Pegtel and Gould, 2019) or be used to identify the phenotypic characteristics and plasticity of their tissue of origin, or to identify biomarkers for these phenotypes. Indeed, blood-derived exosomes have been shown to exhibit significant changes in their RNA composition during lactation and dry periods in dairy cattle (Shi et al., 2023) and play a role in maintaining the normal function of bovine mammary epithelial cells (Wang et al., 2022). Additionally, plasma-derived sEVs may serve as carriers of proteic biomarkers of dairy cow fertility and metabolic status (Almughlliq et al., 2019; Turner et al., 2021; Abeysinghe et al., 2023), heifers fertility (Gad et al., 2020), tick resistance (Abeysinghe et al., 2021; Turner et al., 2022b), and mastitis (Ji et al., 2022). These findings highlight the biological relevance of plasma-derived sEV proteome profiling in cattle and reinforce the interest in future investigations on the links between sEVs cargo composition, physiological status, and productive traits. However, the extraction and purification of sEVs from complex biofluids such as blood plasma presents significant challenges (Takov et al., 2019; Martins et al., 2023; Ter-Ovanesyan et al., 2023), and research on sEVs in livestock animals is only currently emerging. These challenges arise from overlapping size, density, and biophysical properties of sEVs and other mammalian plasma components, such as lipoproteins. Compared to humans, a distinctive characteristic of bovine plasma is its great proportion of high density lipoproteins (John Chapman, 1986; Bauchart et al., 1999; Gruffat-Mouty et al., 1999; Bauchart et al., 2006) which share the same size and density as sEVs (Menard et al., 2018). Consequently, lipoproteins outnumber by 10^3^–10^6^ sEVs (Nieuwland and Siljander, 2024), leading to frequent co-isolation of both particle types (Liangsupree et al., 2021; Lozano‐Andrés et al., 2023; Reymond et al., 2023; Welsh et al., 2024). To address these challenges, various protocols have been developed for sEV extraction and purification (Pegtel and Gould, 2019) principally from human and murine biofluids (Gurunathan et al., 2019; Liangsupree et al., 2021; Chen et al., 2022; Ji et al., 2022; Dilsiz, 2024) such as milk, plasma, urine, and saliva. However, these techniques depend heavily on the biofluid used, which can limit their reproducibility, efficiency, speed, and cost. Furthermore, most methods have been optimized for nonbovine species, despite significant differences in plasma lipoprotein profiles across species (Forte et al., 1981; John Chapman, 1986; Staron et al., 2000). Some protocols were proposed from plasma (Gad et al., 2020; Muroya et al., 2020; Abeysinghe et al., 2023; Shi et al., 2023; Turner et al., 2023) or serum samples (Bok et al., 2024) for Bos species. However, they present limitations, as some of them can be time-consuming, designed for a small number of samples, require substantial plasma volumes (10∼200 mL), and often result in lower purity compared to samples from other mammalian species (Takov et al., 2019). We hypothesized that bovine plasma-derived sEVs could be effectively extracted and purified with great purity using a small plasma volume, as previously demonstrated for mouse (André-Grégoire et al., 2023), rat (Baranyai et al., 2015), and human (Bettio et al., 2023; Nieuwland and Siljander, 2024) plasmas. Our objective was to adapt the most recently proposed protocol in cattle (Turner et al., 2023) for smaller volumes (4 mL) on post-pubertal heifers raised under two different feeding regimes. To ensure compliance with the Minimal Information for Studies of Extracellular Vesicles (MISEV) guidelines of morphological and molecular characterization of sEVs (Théry et al., 2018; Welsh et al., 2024), various complementary techniques were employed.

## Materials and Methods

All procedures involving animals were approved by the ethics committee of the Fribourg canton of Switzerland (2020-45-FR).

We have submitted all relevant data of our experiments to the EV-TRACK knowledgebase (EV-TRACK ID: EV250099) (EV-TRACK Consortium et al., 2017).

**Figure 1.**
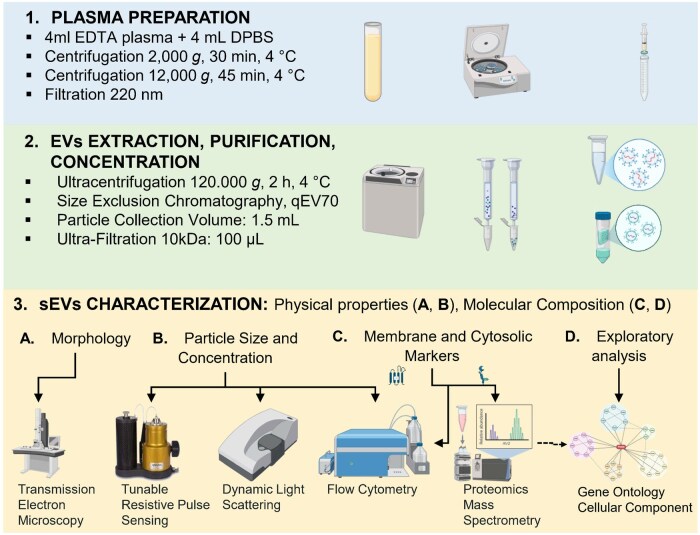
Workflow of plasma-derived sEV methods of preparation, purification, and analysis.

The Figure 1 summarizes the overall workflow for the sEV enrichment process.

### Animals and plasma preparation from bovine blood sample

This study was part of a larger experiment previously described (Xavier et al., 2023; Lindtke et al., 2024), from which we used plasma samples collected from 38 crossbred heifers; dam Brown Swiss (BS), sires Angus (AN), Limousin (LI) or Simmental (SI) reared at the experimental facilities of Agroscope (Posieux, Switzerland). They were fed grass-based diets according to two treatments; with pasture (PA) or with no pasture (NP). The distribution was as follows for PA (BS × AN = 6, BS × LI = 8, BS × SI = 6) an NP (BS × AN = 6, BS × LI = 8, BS × SI = 4). Animals were slaughtered at 530 kg of body weight (SD = 7 kg) with 496 days of age (SD = 28).

Blood samples were collected in 2021 when animals targeted the expected body weight of 530 kg, before feed distribution (6:30–8:30 a.m.; the animals had no access to feed since midnight, before slaughter) to reduce as much as possible the presence of circulating lipoproteins in plasma (Pegtel and Gould, 2019; Nieuwland and Siljander, 2024). Venipunctures using BD Vacutainer^®^ Precision Glide™ needles (18G × 1.5 in.; 1.2 × 38 mm; Becton Dickinson, Franklin Lakes, NJ, USA) from the jugular vein were performed. The blood was drawn into EDTA tubes and immediately placed on ice. Within a maximum of 1.5 h post-collection, the samples were centrifuged at 3,000×*g* for 15 min at 4°C. The plasma was carefully collected, to avoid contamination from the “buffy coat” containing platelets and frozen at −80°C for further use.

All materials and reagents used in this study were purchased from Sigma (Merck, Darmstadt, Germany), unless otherwise specified.

### Extraction, purification and concentration of sEVs

*Plasma dilution and filtration.* Plasma samples were thawed overnight at 4°C and diluted 1:1 with filtered Dulbecco’s Phosphate Buffered Saline (DPBS, pH 7.0–7.2, Panbiotech, Germany, P04-36500). The plasma was centrifuged twice at 4 °C, 2,000×*g* for 30 min, and the supernatant was carefully transferred to a new tube and centrifuged again at 12,000×*g* for 45 min. The resulting supernatant was filtered through a 0.22 µm filter (Merck, Cork, Ireland. Millex-GV, SLGVM33RS) to remove most of the non-vesicular extracellular particles (NVEPs) larger than 220 nm such as chylomicrons, large EVs, or platelets.

*Extraction by ultracentrifugation.* The filtered plasma was transferred into a 12 mL polypropylene ultracentrifuge tube (Beckman Coulter, Brea, USA. 355642). The UC was performed at 120,000×*g* (medium radius) for 2 h at 4°C to pellet sEVs with a 70.1 Ti Fixed-Angle Titanium Rotor (Beckman Coulter). The supernatant was discarded by gentle pouring, and the pellet was resuspended in 500 µL of DPBS overnight.

*Purification through size exclusion chromatography and ultrafiltration.* The day after, the pellets containing sEVs were vortexed (30 s, max speed: 2700 rpm); this is a critical step to efficiently separate any remaining agglomerated particles and maximize sEVs recovery. The sEVs were then loaded onto commercial size-exclusion chromatography (SEC) columns (qEV original/70 nm, Izon Science, Christchurch, New Zealand) specifically designed for biofluids such as plasma. The qEV70 Gen2 columns were used following the manufacturer’s instructions and reused up to 3 times after an appropriate regeneration process (Izon Science Ltd; Izon Science Ltd). Briefly, 500 µL of crude sEVs had been fully loaded onto the qEV70 columns, filtered DPBS was added on top. The first 2 mL of flush, representing the void volume, was discarded, and the subsequent 1.5 mL was collected directly in a single fraction as the particles collection volume (PCV), containing the majority of the sEVs, with minimum lipoprotein contamination. The PCV cut-off has been adjusted based on previous trials involving micro-fractions of 200 µL (SM1), which were collected manually, and for which the tube weights were monitored. The DLS measurements were used to assess each micro-fraction and ensure that the particles were within the expected sEV range. Between each sample run, the qEV70 columns were regenerated by flushing with 8.5 mL of NaOH (0.5 M) solution, followed by 17 mL of filtered DPBS. One 50 µL purified sEVs aliquot was harvested par animal directly from PCV, stored at 4°C and used for size, concentration, and membrane molecular marker analysis using tunable resistive pulse sensing (TRPS), dynamic light scattering (DLS), and nano flow cytometry (FC). Additional 150 µL aliquots from PCVs from each animal were harvested and pooled by groups (breed × husbandry practices) for transmission electron microscopy (TEM) measurements purposes. Resulting PCVs and pools volumes (Each of the six groups has one pool sample.) were reduced by ultra-filtration (UF) following manufacturer instructions using 500 µL Amicon filters with a 10 kDa molecular weight cut-off during three runs of 30 s each to load the entire PCV to eliminate small debris and free proteins (W. Liu et al., 2022) and to concentrate the sEVs (Jia et al., 2022). The necessary volume of DPBS required to reach the expected final volume of 100 µL after UF was used to wash the filters, ensuring the recovery of any residual particles that may have adhered to the 10 kDa regenerated cellulose membrane. Samples were then stored in 0.5 mL Eppendorf^®^ LoBind tubes at −20°C for downstream applications.

### Methods based on physical properties and molecular composition

*Transmission electronic microscopy for sEVs morphology assessment.* The sEVs were visualized by TEM using a negative staining method. The sEVs samples were fixed with a solution containing 2% paraformaldehyde (PFA), 2% glutaraldehyde, and 50 mM cacodylate buffer (pH 7.4) for a duration of 15 min to 1 h. Copper grids with a Formvar/carbon coating (FCF150-Cu, Electron Microscopy Sciences) were used for sample preparation. A 20 µL drop of sEVs sample was placed on a piece of parafilm, and a copper grid (dark side down) was floated on the sample for 10 min at room temperature. After sample adsorption, the grid was transferred to a drop of UranyLess staining solution (Delta Microscopies) previously filtered through a 0.22 µm syringe filter. The grid was incubated face down on the stain for 30 s. Excess stain was removed by gently touching the edge of the grid to a piece of filter paper. The grids were left to air-dry for approximately 15 min on a piece of filter paper before being stored in a grid storage box at room temperature for subsequent analysis. The grids were observed using a transmission electron microscope H-7650 (Hitachi) at 80 kV and a Hamamatsu AMT 40 camera (4k). The images were acquired at various magnifications, with careful adjustment of stage position (X/Y), brightness, and focus. Magnification and focus adjustments were performed using manual knobs, and histogram centering was checked before image acquisition. The sEVs size and morphology were evaluated based on the acquired images for the 6 pools, with a qualitative visual evaluation of the ratio sEVs/lipoproteins like particles. pictures presented on figures are representative of the overall observations performed (n pictures = 142).

*Tunable resistive pulse sensing for sevs particle diameter and exact concentration assessment.* The sEVs were analyzed using TRPS with the qNano Gold instrument (Izon Science, Christchurch, New Zealand) following requirement for sEVs analysis [Maas et al., 2014; Gross-Rother et al., 2020; Izon Science Ltd; Tunable Resistive Pulse Sensing (TRPS) for Exosome Characterization]. The analyses were performed using a 150 nm nanopore membrane (NP150) and Izon Control Suite software. The calibration was carried out using CPC100 calibration particles (nominal diameter 110 nm; Izon Science), diluted 1:2,000 in PBS + 0.03% Tween. Samples were measured at pressures of 7 and 10 mbar, with the nanopore membrane stretched to 47 mm approximately and the applied voltage adjusted to maintain a stable baseline current of approximately 120 mV. Each sample was diluted 1:1 in filtered DPBS + 0.03% Tween-20 to ensure the optimal particle concentration for accurate detection and sizing. The samples were recorded with a minimum particle rate of 100 particles per minute and with at least 500 particles counted. Once the sample were measured, samples were stored at 4°C before DLS and FC analyses. The concentration of particles per mL of plasma volume was calculated as: (PCV concentration in particles per mL × total PCV volume)/total plasma volume used.

*Dynamic light scattering for particle diameter and relative concentration assessment.* Plasma-derived sEVs samples were analyzed using DLS with a Zetasizer Nano ZS (Malvern Panalytical, Palaiseau, France). This technology has previously been recommended for the study of particles such as sEVs in biofluid samples as it allows the estimation of particle hydrodynamic diameter (Bhattacharjee, 2016; Zamani Kouhpanji and Stadler, 2020; Jia et al., 2023; Welsh et al., 2024). The analyses were conducted following international recommendations (ISO 22412 2017; Particle size analysis–DLS). Disposable microcuvettes adapted for size measurements using NIBS (Non-Invasive Back Scatter, 173 degree) were used, in which 40 µL of each sample was introduced. The analyses were conducted at a controlled temperature of 25 °C and triplicated, after 120 s of equilibration. The measurement duration was left on automatic, and for each record the system was allowed to automatically select the optimal number of runs (generally ranging between 12 and 20 per acquisition). The analysis model used was the high resolution multiple narrow modes. Raw data were processed by the software (ZetaSizer Software V8.02), to estimate particles diameter between a 0.4 to 10,000 nm range. The Z-average size and polydispersity index were assessed. The particle size was calculated directly from the variation of intensity of light scattering due to Brownian motion of particles. For conversion of intensity signal to volume and number signal [performed by the software and based on the Mie theory and Rayleigh approximation (Stetefeld et al., 2016)], the viscosity and refractive index of DPBS (the buffer used to dilute sEVs) at 25°C were used following Malvern recommendations (viscosity of 0.8882 cP, refractive index of 1.33). After measurement, the samples were stored at 4°C before FC analysis.

*Nano-FC for particle diameter assessment and CD9 labeling of sEVs.* The FC was performed after TRPS and DLS measurements on the same samples. The FC were performed using a Flow Nanoanalyzer, (NanoFCM Inc., Christchurch, New Zealand) equipped with a 488 nm and a 638 nm laser. The calibration of particle concentration was performed using proprietary standard 250 nm silica calibration beads provided by NanoFCM, following the manufacturer’s guidelines. The calibration of size was performed with monodisperse silica beads of four different diameters (68, 91, 113, and 155 nm) used as standards size reference for NanoFCM with a defined concentration of 2.2 × 10^10^ particles /mL. The FC analysis was performed on the 6 pools of PCV (1 per group). Pools were then completed using 0.22 µm filtered PBS (fPBS) to reach a final volume of 1000 µL. Pools were then incubated with a FITC anti-human CD9 antibody (clone HI9a, BioLegend^®^) at a final concentration of 1 µg/mL, according to the manufacturer’s instructions for cow affinity. The same procedure was carried out for 1000 µL of fPBS for negative control. For the isotype control, a sample pool, prepared by pooling 10 µL from each plasma sample, was incubated with the isotype control at a final concentration of 1 µg/mL. All samples were incubated at RT in the dark for 30 min. They were then transferred to ultracentrifuge tubes and adjusted with 10 mL of fPBS. To remove any unbound antibody, the samples were subjected to another UC (2 h, 120,000×*g* and 4 °C) to pellet sEVs at the bottom of the tube, while leaving unbound antibody particles in the supernatant. The supernatant was then discarded and sEVs pellets were resuspended in 40 µL of filtered fPBS to obtain the appropriate particles concentration for FC. Particle fluorescence was measured in the FITC channel to evaluate labeling efficiency. The FITC-labeled anti-CD9 antibodies were used to stain ­specific membrane proteins on the sEVs. Controls included unstained samples, PBS with antibody-only samples, and isotype FITC antibody-labeled samples to assess nonspecific binding. The output included total particle concentration, size distribution (mean size in nanometers), the percentage of FITC-positive particles, and the concentration of FITC-positive sEVs. Data and plot derived plots were generated from events recorded for 1 min per sample resulting in a particle count superior to 2,000 events, with FITC events gated based on fluorescence intensity, with a sample pressure of 1.0 kPa. Data were analyzed using NanoFCM proprietary software (NF Profession 2.12 offline).

### Protein quantification by BCA and purity estimation

The protein concentration of sEVs in the PCVs was determined using a bicinchoninic acid assay (BCA) Protein Assay Kit (ThermoFisher Scientific, Rockford, USA, Kit 23227) according to the manufacturer’s instructions. The total protein concentration and the corresponding quantities obtained were used to calculate the required sample volumes for proteomic analysis (10 µg per sample). The purity of sEVs preparations was assessed by calculating the particle-to-protein (p:p) ratio, expressed as the total number of particles measured per total µg of proteins assayed, which serves as an indicator of sEVs enrichment. The particle count was derived from the concentration estimated through TRPS measurements, as described above and total proteins estimated from BCA concentrations results.

### Peptide preparation using the S-Trap method

Extracted proteins (10 µg) were prepared using the S-Trap protocol (Protifi, Huntington, NY, USA) following the manufacturer’s recommendations with the subsequent modifications. Briefly, the extracted proteins were dried using a SpeedVac concentrator (45°C, 3 h, full vacuum), to reduce volume and adjust protein concentration. Then the proteins were solubilized in a 4% SDS solution. To reduce disulfide bonds and alkylate the proteins, 20 mM DTT (dithiothréitol) and 50 mM iodoacetamide were added. The samples were then acidified using phosphoric acid (final concentration of 1.2%). The samples were diluted with an S-Trap binding buffer containing 90% ethanol, promoting the formation of protein colloidal particles. The solution was then applied to S-Trap microcolumns and centrifuged (4,000×*g*, 10 min) to trap the proteins onto the modified silica matrix. Once captured, the proteins were washed three times with the binding buffer to remove contaminants. The trapped proteins were digested with trypsin at a 1:50 ratio, corresponding to 0.2 µg of trypsin for 10 µg of proteins in this study. Incubation was carried out overnight at 37°C for a minimum of 16 h to ensure complete digestion. The generated peptides were eluted by 3 successive centrifugations using solutions of 50 mM TEAB, followed by 0.2% formic acid and 50% acetonitrile. Peptides were dried by vacuum evaporation (speed-vac) and resuspended in 20 µL with a solution of isotopically labeled peptides 40 fmol/µL in formic acid 0.1%. The samples were transferred to glass HPLC vials and stored at −20°C until further analysis.

### Mass spectrometry

The peptide mixtures were analyzed by nano-LC-MS/MS (Nanoscale liquid chromatography coupled to tandem mass spectrometry) using a nano-HPLC system (Ultimate 3000, Dionex) coupled to an Orbitrap QExactive HF-X mass spectrometer (Thermo Fisher Scientific). A 2 µL volume of peptide corresponding to 1 µg of total protein extract was first concentrated and desalted at a flow rate of 30 µL/min on a C18 pre-column 5 cm length × 100 µm (Acclaim PepMap 100 C18, 5 µm, 100 Â nanoViper) equilibrated with Trifluoroacetic Acid (TFA) 0.05% in water. After 6 min, this preconcentration column was switched online with an analytical nano-flow C18 column (Acclaim PepMap 100–75 µm inner diameter × 25 cm length; C18–3 µm–100 Å); equilibrated at a flow rate of 400 nL/min with a 96% solvent A (99.9% H_2_O, 0.1% formic acid). Peptide separation was achieved based on hydrophobicity using a gradient of solvent B (99.9% ACN, 0.1% formic acid—vol/vol) from 4% to 25% over 70 min. For MS analysis, eluted peptides were electro sprayed in positive-ion mode at 1.6 kV through a nanoelectrospray ion source heated to 250°C. Mass spectrometry analyses were performed using two different acquisition methods depending whether individual samples or pools samples were analyzed.

(i) Individual sample analysis: A data-dependent acquisition “Top 18” method was employed. The instrument performed a full MS scan followed by 18 data-dependent MS/MS scans triggered by the most abundant ions. Parent ions were selected in the Orbitrap cell (FTMS) at a resolution of 60,000 (IT = 100 ms) and each MS analysis was followed by MS/MS with analysis of the MS/MS fragments with a resolution of 15,000 (IT = 50 ms).

(ii) Pool sample analysis: 1 µL from each animal protein extract were mixed to prepare a pool analyzed in five times at regular intervals to check for signal drift. A “Top 15” data-dependent acquisition method was used, combined with a Parallel Reaction Monitoring method (PRM) (F. Liu et al., 2022; Bezstarosti et al., 2024) targeting peptides from proteins CD9, CD81, HSP70, and TSG101 with the subsequent parameters: MS/MS analysis of the MS/MS fragments with a resolution of 60,000 (IT = 300 ms) and a collision energy (CE) of 28. The Skyline software (v23.1) was employed to generate potential trypsin-digested peptide sequences for bovine proteins CD9, CD81, HSPA8, and TSG101. The sequences of these proteins were imported into Skyline, where a spectral library and a background proteome were created based on the FASTA sequences. Specific precursor and fragment ions were selected for PRM using Skyline’s targeted method editing tools, analysis.

The raw data of the peptide MS/MS spectra were processed using Progenesis QI software (version 4.2, Nonlinear Dynamics, Newcastle upon Tyne, UK). After performing an automatic alignment of all other runs against a reference run defined by the software, the detected ions were grouped in a file (mgf) directly exported to the Mascot Server 3.1 interrogation engine (http://www.matrixscience.com) and searched against a ref_bos_taurus_241029 (59,261 sequences) database with the subsequent parameters: precursor mass tolerance of 10 ppm and fragment mass tolerance of 0.02 Da, a maximum of two missed cleavage sites of trypsin, carbamidomethylation (C), oxidation (M) and deamidation (NQ) set as variable modifications. Proteins were identified with a minimum of two peptides with a false discovery rate (FDR) threshold set to 1% (*P *< 0.02) and a Mascot ion score cutoff of 34.

### Gene ontology and sEV enrichment

Only proteins identified with at least two significant peptide sequences (462) were included in the GO analysis. Proteins flagged as contaminants by the MASCOT software were excluded. Proteins identified as associated with lipoproteins (APO) were excluded from the GO analysis, as their origin is already known and does not require assessment of cellular provenance. This resulted in a final list of 406 proteins submitted for analysis. The search for human homologs was initially performed using ProteINSIDE (Kaspric et al., 2015) based on bovine gene names. When this approach failed, sequence alignments were conducted via BLASTp against the human proteome to retrieve the gene names of the best hits. In cases where multiple hits shared the same e-value, the one with the greatest alignment coverage (on both the human and bovine sequences) was selected. If several hits still tied, all associated gene names were retained. If no alignment met these conditions, no human homolog was assigned to the sequence. Enrichment in GO terms were conducted using ProteINSIDE and ShinyGOV0.82 (Ge et al., 2020), focusing on cellular components (CC) with *P *< 0.01 (FDR). The human genes names corresponding to the identified proteins were compared with several databases, including the Human Blood Plasma Database (Uhlén et al., 2015), a recent proteomic dataset from bovine plasma-derived sEVs (Turner et al., 2023), and Vesiclepedia databases (Chitti et al., 2024).

## Results

The MISEV guidelines (Welsh et al., 2024) were followed in the collation and presentation of all results.

### The sEV shape and morphology assessed by  TEM

**Figure 2.**
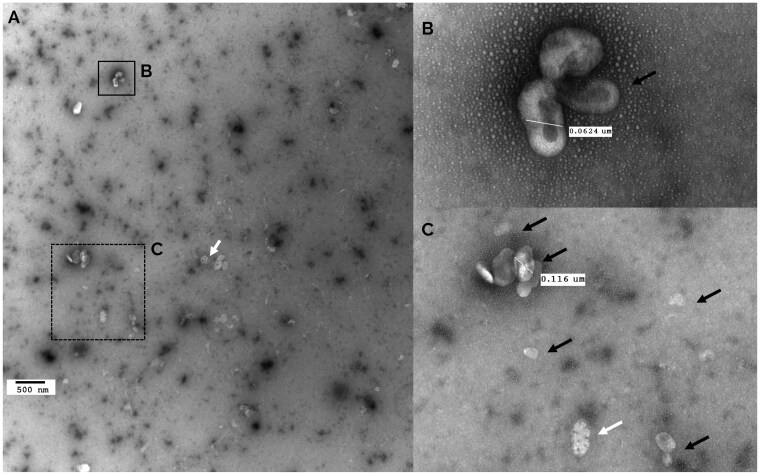
A TEM analysis of sEVs isolated from bovine plasma with negative UranyLess staining. (A) Wide-field view of sEVs showing their morphology. (B) Close-up showing vesicles with the cup-shaped morphology typical of sEVs. (C) Close-up showing a cluster of aggregated particles, a common feature of plasma-derived sEVs. Scale bars are indicated on the pictures of the sEVs. Black arrows indicate particles showing sEVs structures. White arrows indicate particles showing lipoprotein structures.

Particles with the characteristic cup-shaped morphology of sEVs were observed. Few particles displayed lipoprotein-associated morphologies (Figure 2). The size of observed particles fell within the expected sEVs range of 30 to 150 nm.

### Particle diameter, concentration, and protein concentration in PCVs

**Figure 3.**
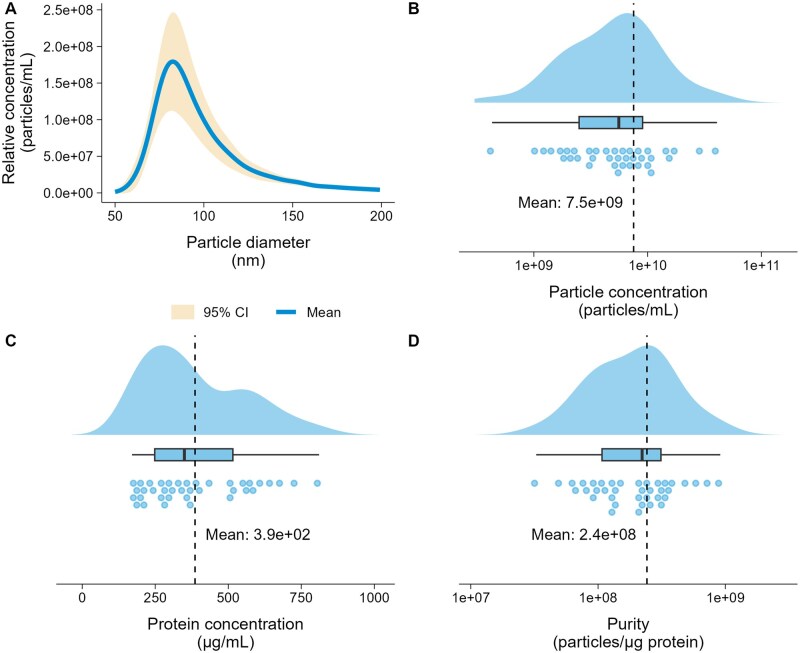
Characterization of bovine sEVs measured by TRPS* and protein concentration, *n* = 38. (A) Particle diameter distribution and relative concentration of sEVs with 95% confidence interval, based on measurements across all animals. (B) Particles concentration in PCV. (C) Protein concentration in concentrated PCV. (D) Purity of sEVs, total number of particles per total µg of protein. (*qNano, 150 nm nanopore, operated at 7 and 10 mbar pressure).

The mean diameter of the particles assessed by TRPS analysis (range assayed 70–400 nm) was 103.9 nm (Table 1). The average concentration was 7.5 × 10^9^ particles/mL of PCV, and of 2.8 × 10^9^ particles/mL of plasma. The protein concentration after the UF step ranged from 171 to 811 µg/mL with a mean of 386 µg/mL. Purity estimation was on average of 2.4 × 10^8^ particles/µg of proteins (Figure 3).

**Table 1.** Particle distribution in nm, categorized by methods.

Method	Sample	N	Min	1st Qu	Median	Mean	3rd Qu	Max	SD	Size range
**TRPS**	Animals	38	50	80	91	103.9	111	572	42.20	70–400
**DLS**			13.50	32.70	37.80	56.27	68.10	1480	47.13	0.4–10.000
**FC**	Pools	6	47.75	64.25	71.25	76.8	83.25	199.25	20.56	30–200

**Figure 4.**
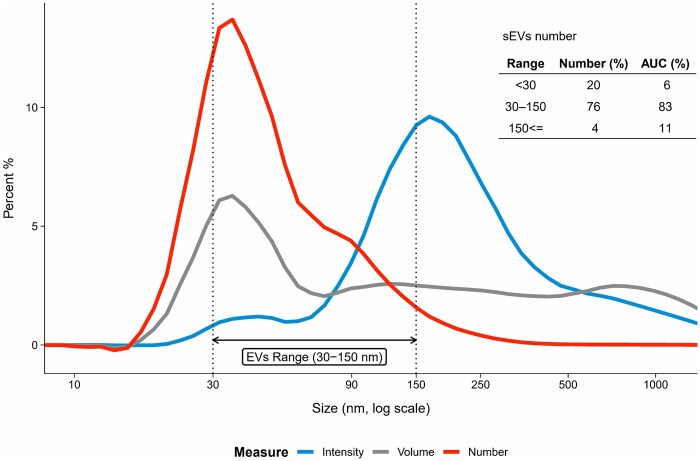
Average dynamic light scattering (DLS) characterization of sEVs isolated from bovine plasma, *n* = 38. The intensity curve (blue line) reflects the measured relative amount of light scattered by particles (scattering intensity) of different sizes, with larger particles highlighted due to their greater scattering. The number curve (red line) shows the calculated relative number of particles, highlighting the predominance of particles within the expected sEVs range of 30–150 nm. The volume curve (grey line) shows the proportional calculated volume occupied by particles of different sizes.

The DLS analysis corroborated TRPS results, with 76% of particles within the sEVs diameter range of 30–150 nm and a mean diameter of 56.3 nm (Table 1, range assayed 0.4–10,000 nm), representing 83% of the area under the curve (AUC). A polydispersity index of 0.464 indicated moderate heterogeneity. Larger particles and aggregates were detected, as shown in intensity and volume curves (Figure 4), as the number curve indicates 4% of total particles above the 150 nm threshold (11% AUC). As expected, the presence of small particles NVEPS was confirmed by the number curve, indicating that 20% of total particles are under the 30 nm threshold (6% AUC).

**Figure 5.**
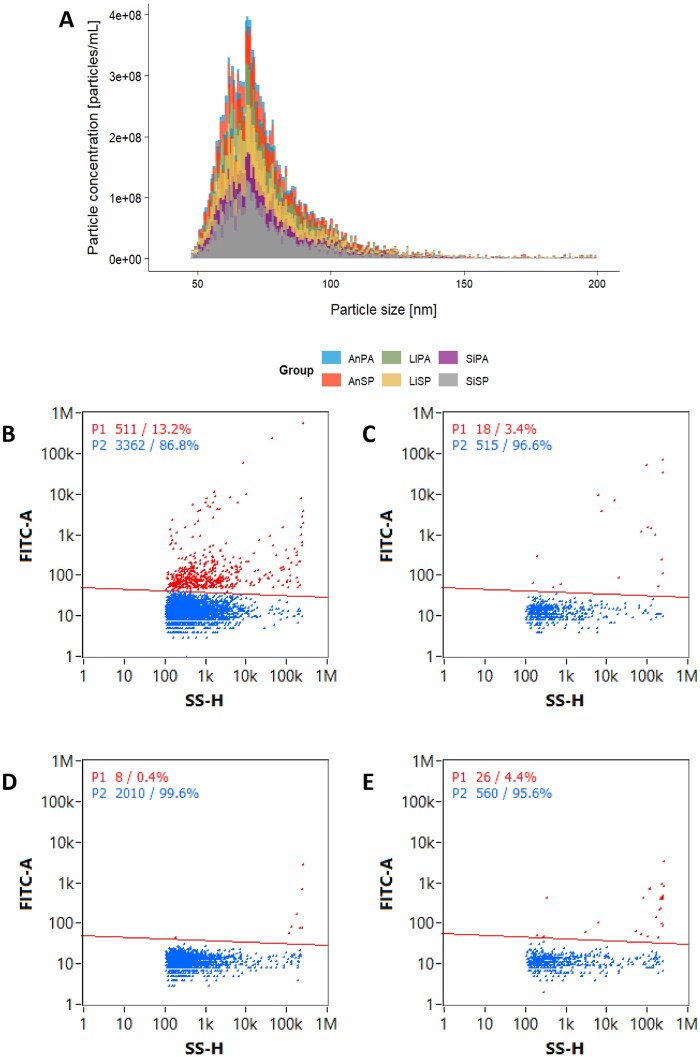
NanoFCM analysis of sEVs samples using FITC-labeled anti-CD9 antibody. (A) Particle size distribution on pooled fractions (B–F). Flow cytometry dot plots showing side scatter height (SS-H, X-axis) versus fluorescence intensity (FITC-A, Y-axis). (B) LI_PA-sEVs pool incubated with FITC-labeled anti-CD9 antibody. (C) Box plot of %FITC+Events for AN_PA-sEVs, SI_PA-sEVs, LI_NP-sEVs, AN_NP-sEVs, SI_NP-sEVs. (D) Isotype control*: LI_PA-sEVs was incubated with FITC-labeled isotype antibody showing low levels of non-specific binding. (E) LI_PA-sEVs without antibody incubation: out of 201 events recorded, only 8 were detected as FITC positive by the laser. (F) Negative control: PBS with FITC-labeled anti-CD9 antibody, showing minimal background fluorescence. FITC-positive (red), unstained (blue).

The FC results (Figure 5A) for particle size distribution indicated that almost the totality of the particles fell within the expected sEVs diameter range as well (mean = 76.8 nm; range assayed 30–200 nm, Table 1), confirming further the TRPS (Figure 3A) and DLS (Figure 4 red curve) results. We nevertheless observed a few positive signals above 150 nm, which is confirmed by each measurement tool used (Table 1).

### Molecular markers of sEVs

*Nano FC.* To detect the presence of the tetraspanin CD9, sEVs pools (Figure 5A) were labeled with a FITC-conjugated anti-CD9 antibody and analyzed using NanoFCM. The percentage of CD9-positive particles ranged from 6.7% to 17.9% across the six pools, confirming the presence of CD9+ sEVs as evidenced by 511 of the 3873 events recorded being detected as FITC positive (Figure 5B, C). Isotype controls and unstained samples exhibited negligible fluorescence, demonstrating antibody specificity. Negative antibody controls (PBS + FITC-labeled anti-CD9) slightly increased event counts, but not in a significant way (Figure 5D–F; Table S1).

*Parallel reaction monitoring and tandem mass spectrometry.* Membrane markers (CD9 and CD81) and cytosolic markers (HSPA8, SDCBP, GAPDH, ACT, and TUB) of sEVs were identified by PRM and/or LC-MS/MS proteomic analysis (Tables S2 and S3). Additionally, sEVs-associated proteins commonly found in vesicles or potentially linked to exomeres or supermeres (e.g., HSPB1, HSP90AA1) were detected. Lipoproteins (APOA1, APOA4, APOB, APOC3, APOC4, APOD, APOE, APOF, APOH) co-isolated with sEVs were also present. All PRM spectra corresponding to sEVs protein markers—including identified peptide sequences, fragment ion matches, and intensity values—are provided in the Supplementary material SM2 and Table S2.

*Proteomic identification and gene ontology enrichment.* The mass spectrometry proteomic data have been deposited to the ProteomeXchange Consortium via the PRIDE partner repository with the dataset identifier PXD068315 and 10.6019/PXD068315.

**Figure 6.**
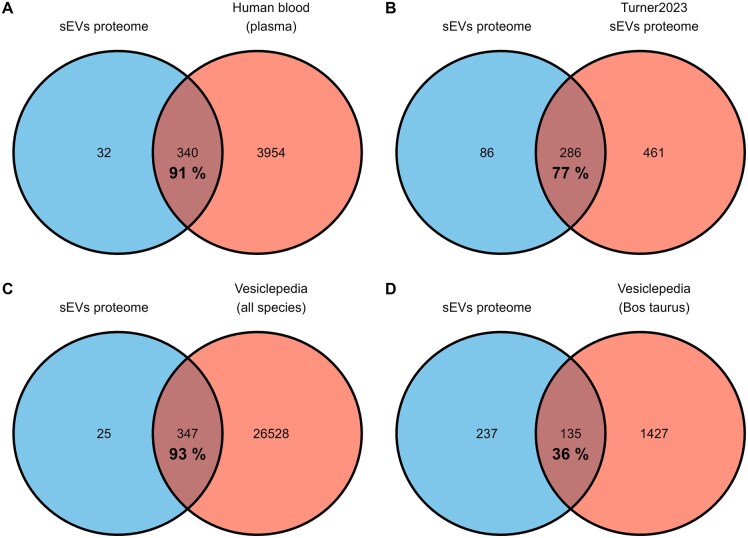
Venn diagrams of proteins detected that match human gene name versus (A) Human protein atlas-blood plasma; (B) bovine plasma derived EVs detected by Turner et al. (2023); (C) Vesiclepedia database 5.1; (D). Vesiclepedia (*Bos taurus*).

The LC-MS/MS proteomic analysis identified 772 bovine accessions (1% FDR), of which 463 with 2 unique peptides corresponding to 417 distinct proteins (Table S3), with 372 of them associated with a human gene name. Among these, 340 gene names (91%) overlap with the human protein atlas database in the “Protein detected in human plasma by mass spectrometry” (Figure 6). Additionally, 286 gene names (77%) have previously been reported in the literature as being associated with plasma-derived cattle sEVs. According to the Vesiclepedia proteome databases, 347 gene names (93%) were found to be associated with sEVs across all species, while only 135 (36%) were specific to *Bos Taurus*. Albumin, the most abundant (60%) free protein in raw plasma accounted for 1.8%–6.5% of the final total protein abundance. Lipoproteins accounted for 4.3%–7.0%. The common platelet activation markers, were not detected except for THBS1.

**Figure 7.**
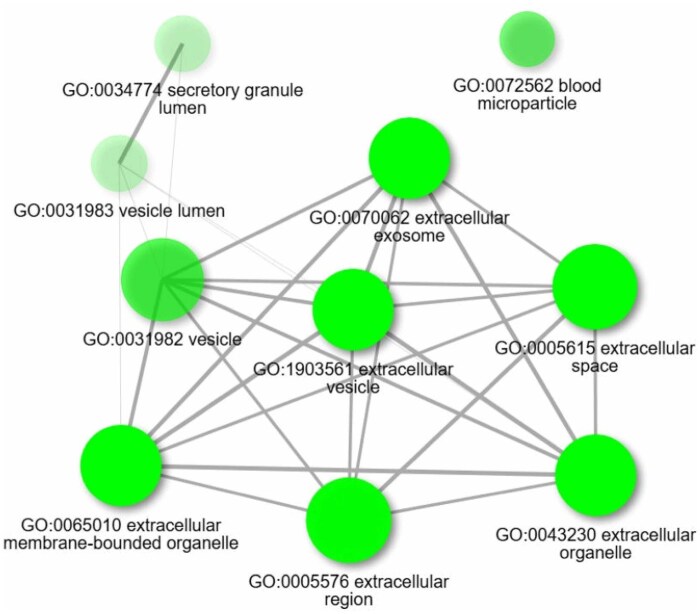
Network visualization illustrating relationships among the top 10 significantly enriched GO cellular component (GO:CC) terms (FDR < 0.01) generated thanks to ShinyGO. Analysis parameters included pathway size limits (min = 2, max = 5000). Nodes represent enriched GO term. The thickness of the lines reflects the percentage of overlapping genes. Node size corresponds to the number of genes associated with each term.

The GO terms enrichment within the CC category (GO:CC) based on human species revealed that identified proteins were predominantly associated with extracellular compartments and vesicular structures (Table S4, Figure S1; Figure 7).

**Figure 8.**
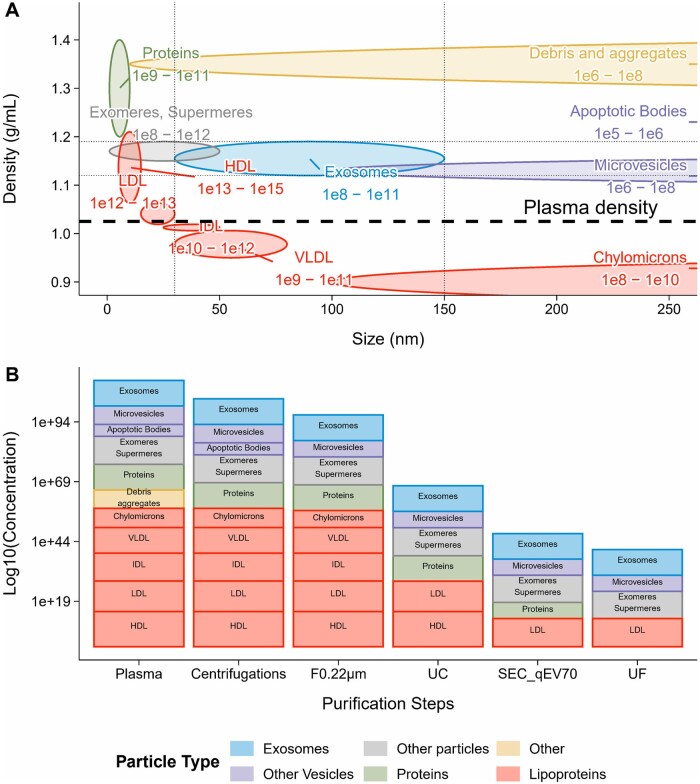
Overview of particle types in mammalian plasma and the impact of purification steps on their populations. (A) Expected concentration ranges, sizes, and densities of plasma particles for each particle type are indicated*. (B) Expected log10(concentration) of particle populations at each step of the purification process. *These values are rough estimates and should be interpreted with caution. Lipoproteins are commonly assumed to be approximately 10³–10^6^ times more concentrated than sEVs in plasma (Simonsen, 2017; Johnsen et al., 2019; Ballantyne, 2024; Chou et al., 2024; Nieuwland and Siljander, 2024).

## Discussion

The main achievement of our protocol is the ability to extract purified sEVs from 4 mL of conserved bovine plasma, with a yield of material available for sEV characterization according to the MISEV guidelines, as well as for proteomic analysis of sEVs. This was performed by using a recent protocol (Turner et al., 2023) originally designed for 20–40 mL of plasma optimized for 4 mL of plasma. One of the original features of this study is its combination of several methods (TRPS, DLS, and FC) for the analysis of the particle size distribution, and the use of PRM mass spectrometry to assess the presence of specific sEV markers. Our previous trials using the originate protocol on small volumes resulted mainly in the isolation of particles under 30 nm (DLS analysis, data not shown). To address this issue, we propose several methodological adjustments to effectively target sEVs. These steps were also suggested by a recent paper (Bok et al., 2024) to increase the yield and the purity of the sEVs starting from larger volumes. As ultracentrifugation (UC) produces a very sticky pellet of particles, particular care was taken during pellet resuspension and homogenization to maximize the yield of small extracellular vesicles (sEVs), as has been recommended in recent studies (Bok et al., 2024). It is crucial to dissociate the particles aggregated during UC as much as possible; otherwise, these aggregates will remain trapped at the top of SEC columns, or agglomerated particles will be eluted too rapidly, because SEC columns are designed to separate single particles.

To achieve this, the pellets were resuspended for at least 12 h before the loading on SEC, and then vortexed 30 s, a duration extended 10 times compared with preliminary tests. The second main modification was the optimization of the PCV collected to recover as many sEVs as possible, while reducing the supernumeration caused by the co-isolation of sEVs with NVEPs or lipoproteins. This is of prime importance for bovine plasma, because HDL particles, which have a density similar to that of sEVs (Figure 8), are present at a greater concentration in bovine plasma than in human plasma (John Chapman, 1986; Gruffat-Mouty et al., 1999; Bauchart et al., 2006).

The PCV of 1.5 mL was adjusted thanks to DLS that enables scanning of a range from 0.4 to 10,000 nm, making it suitable to visualize each particle population according to their sizes (Szatanek et al., 2017; Almughlliq et al., 2019; Serrano-Pertierra et al., 2019). These modifications resulted in a sEV concentration of 7.5 × 10^9^ particles/mL in PCV, which is similar to the concentrations reported by Turner et al. (2023) and Bok et al. (2024), at 2 × 10^9^ and 2.3 × 10^9^ particles/mL, respectively. However, when the results were reported to the volume of plasma used, our protocol resulted in a sEV concentration of 2.8 × 10^9^ particles/mL of plasma. This is greater than the concentration reported in the plasma (1 × 10^8^ particles/mL) of 27-month-old primiparous or 10-month-old Holstein heifers (1 × 10^8^ and 3 × 10^7^ particles/mL respectively, Turner et al., 2023) or in the serum (3 × 10^7^ particles/mL) of Holstein cows (Bok et al., 2024). It should be noted that the few available results were obtained from different biofluids (plasma or serum) and cows under various experimental conditions. Therefore, it remains difficult to ascertain whether the difference in the yield of collected particles was due to technical variations or animal peculiarities. Furthermore, it is interesting to note a similar protein concentration (390 µg/mL in PCV after UF) in our study when compared to those reported by Turner et al (2023) [10–150 µg/mL], and Bok et al (2024) [9.2 µg/mL], the greatest protein concentration has been obtained in F1 across the studies. The total protein yield in our concentrated PCV was 39 ± 17 µg, which was sufficient for downstream mass spectrometry analysis, but not enough to perform western blotting.

Ensuring sEV purity is a major concern during their isolation process and purification. However, no consensus currently exists regarding acceptable standards for this criterium. The p: p ratio is 1 metric used that accounts both the number of sEVs and the amount of sEV-associated proteins (Webber and Clayton, 2013). To obtain reliable sEV size distribution assessment and concentration, it is recommended to combine several methods (Kandimalla et al., 2021). The results from DLS, TRPS and FC were combined, to provide a consistent particle size distribution. However, 20% of particles were found to be smaller (below 30 nm) than sEVs (Figure 4), and may be exomeres, supermeres or lipoproteins (IDL, LDL, and HDL) that outnumber sEVs and affect the p: p ratio. Consistently, TEM analysis showed co-isolation of sEVs, the main particles observed, with some NVEPs particles likely to be lipoproteins. This co-isolation may be due to the fact that lipoproteins are 10^3^–10^6^ times more abundant than sEVs in raw mammalian plasma (Simonsen, 2017; Johnsen et al., 2019; Nieuwland and Siljander, 2024). Our results are consistent with the difficulty of completely removing contaminant particles previously reported (Yuana et al., 2014; Takov et al., 2019; Liangsupree et al., 2021; Lozano‐Andrés et al., 2023; Nieuwland and Siljander, 2024). In addition to the p:p assay, performing a reliable protein quantification may be challenging for sEVs with poor protein content. To counter this, an UF step was added to concentrate the sEVs. Such step has already been proven to be efficient in sEV isolation from plasma (Diaz et al., 2018; Kornilov et al., 2018). The resulting average purity of sEVs in the present study (2.4 × 10^8^ particles/µg protein) was greater than that reported by Turner et al (2023) (3 × 10^7^ particles/µg protein) and similar to that reported by Bok et al (2024) (3 × 10^8^ particles/µg protein). However, it is unclear whether the variations in purity originate from differences among the animals or from technical modifications. Studies in other species, such as rats or humans, showed a wide range (10^4^–10^10^) of purity depending on the extraction protocols and biofluids (Soares Martins et al., 2018; Takov et al., 2019; Li et al., 2022; Ciftci et al., 2023). Complementary purity metrics including protein:lipid ratio and RNA:particle ratio (Théry et al., 2018) would help to better assay the purity of sEVs preparations. Our proteomic analysis detected only the platelet activation marker THBS1, while no other platelet markers were identified. This suggests that the precautions applied during plasma collection and sEV isolation likely limited contamination from platelet-derived sEV. Further reduction in contamination could nevertheless be achieved by implementing the most recent recommendations (Nieuwland and Siljander, 2024). Because purity is a major concern in sEV studies, removing free proteins is crucial. In plasma, albumin accounts for around 60% of total proteins (Barbosa et al., 2010), whereas in our MS results (Table S3) we achieved a 90%–98% depletion, which suggests a great purity of our preparations. Furthermore, according to MISEV2024, albumin cannot be completely removed, and its status as a true contaminant remains debated.

The molecular characterization further confirmed the isolation of greatly pure sEVs. The molecular sEV markers (Théry et al., 2018; Welsh et al., 2024) were detected by mass spectrometry (membrane: CD9, CD81; cytosolic: HSPA8, SDCBP, ACT, TUB, GAPDH) and FC (CD9). The low apparent labeling rate for CD9 (7%–18% after correction, see Table S1) in our study falls within the range of values reported in the literature (Tian et al., 2020; Karimi et al., 2022), suggesting that not all sEVs express CD9. The TSG101 marker was not detected, even by PRM mass spectrometry either, in line with previous studies in bovine plasma (Turner et al., 2022a, 2022b, 2023) but in contrast to the identification of TSG101 in fractions F1 to F4 by Bok et al. (2024) using Western blot. This highlights the variability of sEV markers across fluids and species. In addition, the proteomic analysis revealed a great degree of overlap between the identified proteins and the protein databases of sEVs such as Vesiclepedia (93%), indicating an enrichment of plasma-derived sEV proteins. This is consistent with the results of previous proteomic studies on plasma-derived sEVs, which have shown that combining UC and SEC methods improves the detection of sEV proteins (Alameldin et al., 2021; Turner et al., 2022b). The Gene Ontology analysis of the proteome showed an enrichment of GO terms associated with sEVs, intracellular vesicles (GO:0034774, secretory granule lumen and GO:0060205, cytoplasmic vesicle lumen) and NVEPs (GO:0072562, blood microparticles). Therefore, some intracellular vesicular proteins, potentially involved in sEV biogenesis, may remain within the sEVs after their release (Lozano‐Andrés et al., 2023; Shi et al., 2023), and minor contaminations may persist from NVEPs. This finding validates the effectiveness of our protocol for investigating the proteome of bovine plasma-derived sEVs.

Concerning future perspectives, some authors have proposed a more holistic vision, viewing sEVs and lipoproteins as a continuum of small circulating particles. This interesting viewpoint could ultimately facilitate the discovery of relevant biomarkers (Johnsen et al., 2019). Moreover, because the plasma composition may differ from a species to another, the methods employed may not be fully transposable between species. Similarly, many factors may affect sEV yield and purity within the same species, including the biofluid used (plasma versus serum), breed, sex, physiological stage, and husbandry practices. In the field of animal livestock, a like-for-like comparison of different methods to extract and purify sEVs would be useful as would a comparison with raw proteomes from blood plasma to justify the interest of sEV enrichment in blood plasma for proteomics and biomarker research.

## Conclusion and Perspectives

Our study demonstrates the feasibility of isolating bovine plasma-derived sEVs from small plasma volumes with a great yield and a great purity. The use of several methods (TEM, TRPS, DLS, FC) confirmed that particle size and distribution is consistent with common expected sEVs diameter range (30–150 nm), and the GO:CC analysis proves that most of the extracted proteins belong to the extracellular environment. Although the purity level is comparable to that of other mammalian species, further improvement could enhance sEVs separation from NVEPs in complex media, such as blood plasma, and reduce the volume of cattle plasma required for proteomic analysis. Novel techniques based on structural differences between sEVs and NVEPs (bilayer versus monolayer membrane) like combining different chromatography methods (e.g., combining different resins) may help to tackle such challenge(s). Dual-mode chromatography, or hydrophobic interaction chromatography, chemically destroying lipoproteins using styrene-maleic acid, acoustofluidics properties, magnetic bead, or PEG‐3 precipitation are promising approaches that should be explored in the future.

## Supplementary Material

skaf354_Supplementary_Data

## Abbreviations:

DLS: dynamic light scattering
sEVs: small extracellular vesicles
FC: flow cytometry
FITC: fluorescein isothiocyanate
GO:CC: gene ontology cellular component
[H, L, I, VL]-DL: [high low, intermediate, very low]-density lipoprotein
LC-MS/MS: liquid chromatography–tandem mass-spectrometry
MISEV: Minimal information for studies of extracellular vesicles
NVEP: non-vesicular extracellular particle
PCV: particle collection volume
PRM: parallel reaction monitoring
SEC: size exclusion chromatography
TEM: transmission electron microscopy
TRPS: tunable resistive pulse sensing
UF: UltraFiltration
UC: UltraCentrifugation
