# Correction to: Efficient enrichment of plasma-derived extracellular vesicles from small volumes of bovine blood

**DOI:** 10.1093/jas/skag170

**Published:** 2026-06-10

This is a correction to: Vincent Prieur, Isabelle Cassar-Malek, Arnaud Delavaud, Liam Barry-Carroll, Jean-Christophe Delpech, Isabelle Morel, Sylvain Lerch, Didier Viala, Philip Chennell, Céline Boby, Muriel Bonnet, Efficient enrichment of plasma-derived extracellular vesicles from small volumes of bovine blood, *Journal of Animal Science*, Volume 103, 2025, skaf354, https://doi.org/10.1093/jas/skaf354

In the originally published version of this manuscript, forenames and surnames were incorrectly ordered for all authors with the exception of the corresponding author.

These authors’ names have now been corrected to read: Isabelle Cassar-Malek, Arnaud Delavaud, Liam Barry-Carroll, Jean-Christophe Delpech, Isabelle Morel, Sylvain Lerch, Didier Viala, Philip Chennell, Céline Boby, Muriel Bonnet.

