# Supplementary material for: Efficient enrichment of plasma-derived extracellular vesicles from small volumes of bovine blood
Source: J Anim Sci. 2025 Oct 15;103:skaf354. doi: 10.1093/jas/skaf354 (PMC12597144; doi:10.1093/jas/skaf354)
Supplement: skaf354_Supplementary_Data [file skaf354_supplementary_data.zip › SM1_DLS_pretests.docx]

| Zetasizer pre-test results | | | | | |
| --- | --- | --- | --- | --- | --- |
| Fractions. | Elution volume (mL) | Test 1 | Test 2 | Test 3 | Test 4 |
| sample Volume | 0,5 | discarded | | | |
| Void Volume | 1,5 |  |  |  |  |
| µf1* | 1,671428571 | no | no | no | no |
| µf2 | 1,842857143 | no | good_low_cc | no | good_low_cc |
| µf3 | 2,014285714 | good | good | good_low_cc | good |
| µf4 | 2,185714286 | good | good | good_low_cc | good |
| µf5 | 2,357142857 | good | good | good_low_cc | good |
| µf6 | 2,528571429 | good | good | good | good |
| µf7 | 2,7 | good | good | good | good |
| µf8 | 2,871428571 | good | good | good | good |
| µf9 | 3,042857143 | good | good | good | good_+_small |
| µf10 | 3,214285714 | good_+_small | good_+_small | good_+_small | good |
| µf11 | 3,385714286 | good_+_small | good_+_small | good_+_small | good_+_small |
| µf12 | 3,557142857 | too_small | too_small | too_small | too_small |
| µf13 | 3,728571429 | too_small | no | no | no |
| µf14 | 3,9 | no | no | no | no |
| free proteins | 7,5 | discarded | | | |
| *µf = µ fractions of 3 drops. | | | | | |
| no = no particles detected. good_low_cc = few particles detected at the good size. good_+_small = good particles + small particles detected. too_small = population of small particles outnumbered others particles | | | | | |
