# Supplementary figures and images for: Efficient enrichment of plasma-derived extracellular vesicles from small volumes of bovine blood
Source: J Anim Sci. 2025 Oct 15;103:skaf354. doi: 10.1093/jas/skaf354 (PMC12597144; doi:10.1093/jas/skaf354)

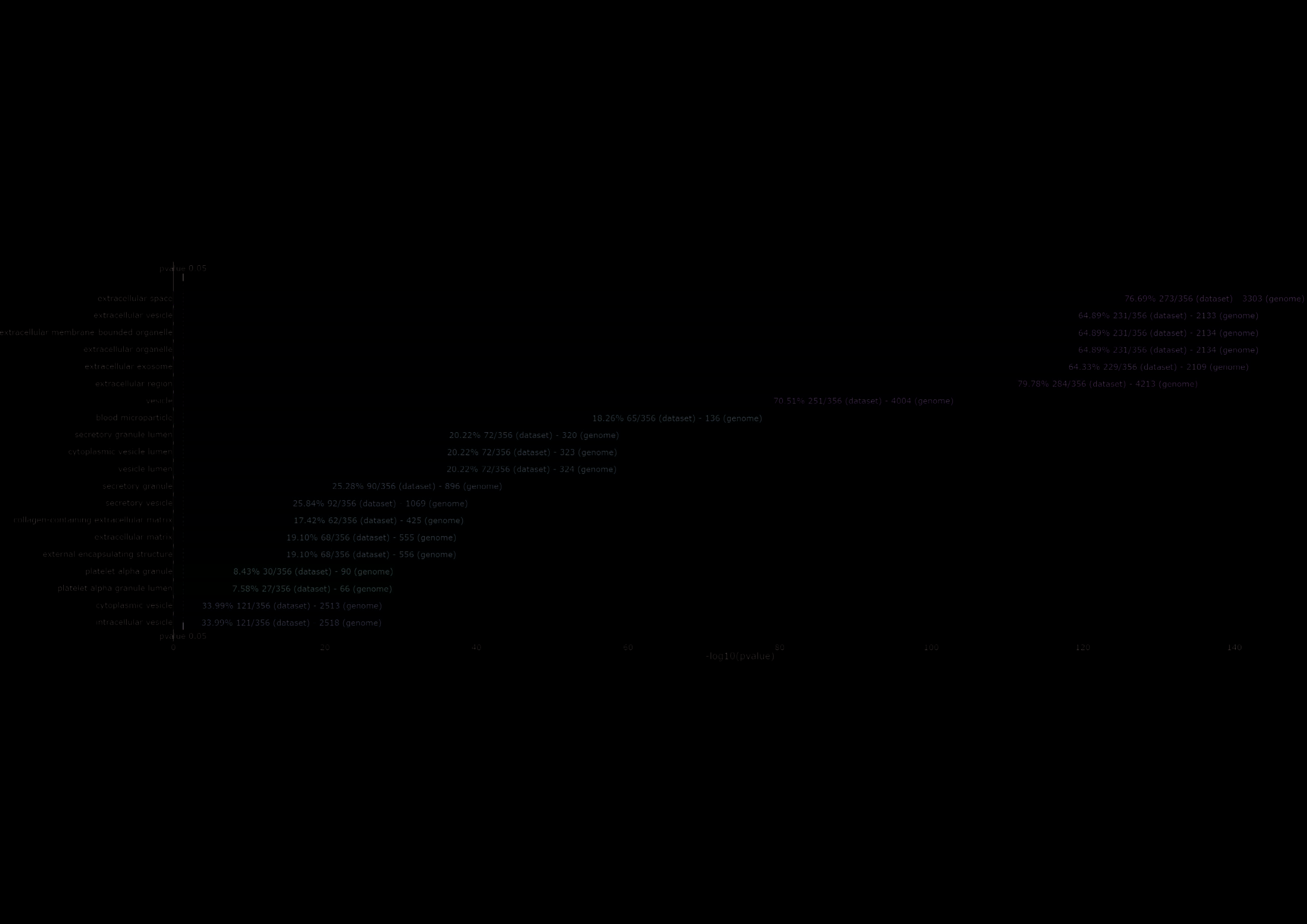

Supplement: skaf354_Supplementary_Data [file skaf354_supplementary_data.zip › FigureS1_ProteInside_GOCC_noAPO_allGO_012025.tiff]
